# The Protective Effect of *Myristica fragrans Houtt.* Extracts Against Obesity and Inflammation by Regulating Free Fatty Acids Metabolism in Nonalcoholic Fatty Liver Disease

**DOI:** 10.3390/nu12092507

**Published:** 2020-08-19

**Authors:** Wenyu Zhao, Fanfen Song, Diangeng Hu, Haiqin Chen, Qixiao Zhai, Wenwei Lu, Jianxin Zhao, Hao Zhang, Wei Chen, Zhennan Gu, Gang Wang

**Affiliations:** 1 State Key Laboratory of Food Science and Technology, Jiangnan University, Wuxi 214122, China; zwyjiangnan@163.com (W.Z.); 17851300257@163.com (F.S.); 7160112050@vip.jiangnan.edu.cn (D.H.); haiqinchen@jiangnan.edu.cn (H.C.); zhaiqixiao@jiangnan.edu.cn (Q.Z.); luwenwei@jiangnan.edu.cn (W.L.); zhaojianxin@jiangnan.edu.cn (J.Z.); zhanghao@jiangnan.edu.cn (H.Z.); chenwei66@jiangnan.edu.cn (W.C.); zhennangu@jiangnan.edu.cn (Z.G.); 2 School of Food Science and Technology, Jiangnan University, Wuxi 214122, China; 3 International Joint Research Laboratory for Probiotics, Jiangnan University, Wuxi 214122, China; 4 (Yangzhou) Institute of Food Biotechnology, Jiangnan University, Yangzhou 225004, China; 5 National Engineering Research Center for Functional Food, Jiangnan University, Wuxi 214122, China; 6 Wuxi Translational Medicine Research Center and Jiangsu Translational Medicine Research Institute Wuxi Branch, Wuxi 214122, China; 7 Beijing Innovation Centre of Food Nutrition and Human Health, Beijing Technology and Business University (BTBU), Beijing 100048, China

**Keywords:** nonalcoholic fatty liver disease, free fatty acids, alcohol extract of nutmeg, obesity, inflammation

## Abstract

Nonalcoholic fatty liver disease (NAFLD) is a disorder characterized by the excess accumulation of fat in the hepatocytes. It is commonly associated with severe obesity and inflammation. Free fatty acids (FFAs) are the key to regulate lipid metabolism and immune response in hepatocyte cells. This study examined the effects of AEN (alcohol extract of nutmeg, the seed of *Myristica fragrans Houtt*.) on the inhibition of lipid synthesis and inflammation in vitro and in vivo and on high-fat diet-induced obesity in NAFLD mice. Our results showed that AEN treatment could downregulate the expression of lipid synthesis-related genes fatty acid synthase (FASN) and sterol regulatory element-binding protein 1c (SREBP-1c) and lower the lipid content of cells. AEN also inhibited FFAs-mediated inflammation-related cytokines interleukin-6 (IL-6) and tumor necrosis factor α (TNFα) expression in cells. In a mouse model, AEN reduced the bodyweight of obese mice and improved NAFLD without affecting food intake. Further analysis revealed that AEN significantly reduced inflammation level, cholesterol and lipid accumulation, blood glucose, and other liver function indexes in mice fed with a high-fat diet. In conclusion, AEN inhibited the aggravation of obesity and inflammation by downregulating lipid-gene expression in the liver to ameliorate NAFLD.

## 1. Introduction

Nonalcoholic fatty liver disease (NAFLD) is one of the important causes of global liver disease [1], which is characterized by diffuse hepatocytes with hepatocellular ballooning, intrahepatic inflammation, and progressive fibrosis [2,3,4]. NAFLD progression can be categorized into simple steatosis (SS), nonalcoholic steatohepatitis (NASH) and fatty cirrhosis, cirrhosis, and even liver cancer [5,6]. The mechanisms of NAFLD progression are widely believed to have at least two components. Fat accumulation caused by lipid metabolism disorder is a common mechanism in hepatic steatosis, followed by immune cell activation and pro-inflammatory cytokine production [3,7]. Studies have shown that obese patients with NAFLD showed decreased lipid metabolism but increased fatty acid biosynthesis [8].The increase of lipolytic activity in adipose tissue results in an increased rate of fatty acid release into the plasma pool. Excess free fatty acids (FFAs) contribute to triglyceride (TG) secretion in the form of very-low-density lipoprotein (VLDL) and increase de novo lipogenesis in the liver [9]. The sustained excess in a fatty acid is not only involved in the pathogenesis of steatosis but also leads to a related inflammatory reaction. The promotion of lipid accumulation within hepatocytes induces sustained hepatic generation of pro-inflammatory cytokines [10]. Interleukin-6(IL-6) concentrations are positively correlated with obesity, impaired glucose tolerance, and insulin resistance [11,12]. Tumor necrosis factor α (TNF-α) increases adipocyte lipolysis to released FFAs. Conversely, FFAs are also significant factors, leading to inflammatory cytokines in adipocytes [13]. 

The fatty acid synthase gene (FASN) plays an important role in fatty acid metabolism, and the inhibition of FASN leads to a decreasing activity of fatty acid synthesis pathways [14]. Given the key role of FASN in catalyzing the synthesis of long-chain fatty acids from malonyl-CoA and acetyl-CoA (ACC), it has become an attractive target for intervention in NAFLD progression caused by excessive lipid accumulation [15]. FASN is regulated by the sterol regulatory element-binding protein-1c (SREBP-1c) [16], one of the SREBP family of cholesterol and lipid synthesizers [9]. In the process of hepatic lipid synthesis, SREBP-1c, as a homodimer, is transported from the endoplasmic reticulum (ER) to the nucleus, where it binds to the SRE sequence and stimulates the transcription of downstream target genes, such as FASN, to regulate fatty acid metabolism [10]. Inhibition of FASN and SREBP-1c expression has been reported to effectively prevent fatty acid biosynthesis, accelerate fatty acid oxidation to abolish the abnormal accumulation of FFAs, and thereby alleviate obesity and other lipid metabolism diseases [8]. As one of the upstream targets of SREBP-1c, AMP-activated protein kinase (AMPK) is closely related to regulating energy balance. The activation of AMPK leads to a decrease in ATP consumption and an increase in ATP production [17,18]. AMPK is phosphorylated to inhibit the expression of the SREBP-1c gene, and thus represses hepatic steatosis [19,20].

Many edible plants have been reported to inhibit lipid accumulation and inflammation. Curcumin ameliorates hepatic steatosis induced by free fatty acids (FFAs), reduces inflammatory response, and treats hepatic fibrosis [21,22,23]. Our laboratory has found that clove extracts can prevent obesity in mice by depressing FASN [24]. Nutmeg (the seed of *Myristica fragrans Houtt.*) is a natural kitchen condiment that has been used for centuries. As a plant seed, fat content in nutmeg ranges from 28 to 38%, and myristic acid is the main part of fat. Phenylalanine is the dominant amino acid in *Myristica fragrans Houtt.* [25,26]. So, it can be added to baked food to strengthen the nutritional effects for phenylalanine that is one of the essential amino acids in the human body. Originally, it was found to be useful in the treatment of gastrointestinal and renal diseases [27]. Later, nutmeg has been reported to have antioxidant, anti-tumor, and antibacterial effects, and more [28,29,30,31,32]. Although nutmeg has a long history of abuse [33,34], and even higher doses (20–80 g of powder) have been ingested (adult), a life-threatening situation has never been observed [35]. In recent studies, nutmeg has displayed high binding activity to the cannabinoid receptor 1 (CB1), which is associated with the obesity pathway [36]. Nguyen et al. found that AMPK activators from nutmeg could inhibit obesity [37]. Oral administration of cinnamon, nutmeg, and peppermint has an anti-diabetic effect in Wistar Albino Rats [38]. Myristicin, an ingredient in nutmeg, has anti-inflammatory properties related to its inhibition of NO, cytokines, etc. [39]. A blend containing *Myristica fragrans Houtt.* can protect acetaminophen (APAP) and carbon tetrachloride (CCl4)-induced acute liver toxicity models in mice [40]. It has also been reported that nutmeg extract could effectively protect against thioacetamide (TAA)-induced acute liver injury via the peroxisome proliferators-activated receptor α (PPARα) signaling pathway [41]. To date, there are few studies to nutmeg extracts, which have targeted on FFA-induced hepatic steatosis and inflammation to relieve NAFLD. In this study, based on data from screening edible plants available in our laboratory [24], we aimed to determine whether the alcohol extract of nutmeg (AEN) is an active ingredient to ameliorate hepatic steatosis and inflammation with the effect of regulating the AMPK-mediated SREBP signaling pathway in mice.

## 2. Materials and Methods

### 2.1. Nutmeg Extract Preparation and Function Identification

The nutmeg (*Myristica fragrans*) was purchased from Tong Ren Tang, Beijing (No. 270028428). The nutmeg seed was crushed and ground by a mechanical grinder and extracted three times with 70% ethanol at 37 °C for 12 h each time. The extracts were combined and filtered by vacuum filtration. The filtered liquid was concentrated in a rotary evaporator, and the solid powder was dissolved in 100% dimethyl sulfoxide (DMSO) (Thermo Fisher, Waltham, MA, USA) or 0.5% sodium carboxymethyl cellulose (CMC) (Sinopharm Chemical Reagent Co., Ltd., Shanghai, China) for in vitro (DMSO) and in vivo (CMC) experiments separately. Following the screening method described in previous articles, AEN was screened as a lipid synthesis inhibitor by *Mortierella alpina* ATCC32222 (MA) in 96 well plates [24]. Each group was prepared in triplicate. The control group (MA) received only the solvent (DMSO). The mold was placed in an incubator at 28 °C for 72 h in darkness, and the color change was observed.

AEN samples processed were detected by GGT 0620 comprehensive two-dimensional gas chromatography (GC × GC)-time-of-flight mass spectrometer (TOF-MS) (Guangzhou Hexin Instrument Co., Ltd., Guangzhou, China). The following test conditions were GC × GC-TOF-MS methods. (a) GC conditions: The temperature of the injection port was 280 °C, and the first dimension chromatographic column was db-5ms (30 m × 0.25 μm). The second-dimensional column was db-17 (1 m × 0.18 mm × 0.18 μm). The carrier gas was helium, with a constant current mode control and a column flow of 1 mL/min. The start temperature of the column box was 40°C; it was kept for 1 min, warmed to 300 °C for 6 min, kept for 5 min, making it a total of 49.33 min. (b) Full 2D gas-phase modulator condition: The modulation column was an HV modulation column (1.1 m × 0.25 mm) with a modulation period of 4 s. (c) Time of flight mass spectrometry detector conditions: The ion source temperature was 230 °C, the interface temperature was 280 °C, the detector voltage was −1750 V, the ionization energy was 70 eV, the acquisition mass range was 35–550 amu, the acquisition speed was 100 spectra/second, and the acquisition time was 3–49.33 min.

### 2.2. Cell Lines and Viability Assays

The human hepatocyte line LO2 and mice macrophages RAW 264.7 cells were cultured in Dulbecco’s Modified Eagle Medium (Thermo Fisher, Waltham, MA, USA) containing 10% FBS (Thermo Fisher, Waltham, MA, USA) plus 100 U/mL penicillin and 100 μg/mL streptomycin. LO2 and RAW 264.7 cells were plated in 96-well plates at a density of 5000 cells per well. After 24 h, the cells were treated with different concentrations of nutmeg extract in four dose groups (25, 50, 100, and 200 µg/mL). The control cells received only the solvent (DMSO). Each group was repeated in triplicate. After 72 h, cell proliferation was measured using the thiazolyl blue tetrazolium bromide (MTT) (Sigma, St, Louis, MO, USA) assay. The culture medium was discarded, and the cells were carefully rinsed three times with PBS, after which the culture medium containing MTT was added and left for 4 h. Finally, DMSO was added to dissolve the bluish-violet crystals (formazan) in the cells. The cell viability was measured by absorption at the 570 nm wavelength in a microplate reader.

### 2.3. Animal Treatment

Thirty-four-week-old male C57BL/6/J mice of SPF grade were purchased from Grace (Shanghai, China). The experimental protocol was approved by the animal ethics committee of the Jiangnan University of China and followed the European Community code of ethics guidelines (Directive 2010/63/EU). The experiment was carried out in the animal center of Jiangnan University (JN. No 20180115c0800815). After one week of acclimatization and 12 short dark cycles in a pathogen-free, temperature-controlled environment, the mice were randomly divided into three groups (ten mice in each group): one normal diet group (10% fat diet, Beijing HFK Bioscience Co., Ltd., Beijing, China) and two high-fat diet groups (60% fat diet, Beijing HFK Bioscience Co., Ltd.). Methods referenced to the instruction of D12492 rodent diet containing 60 kcal% fat (Research Diets, Inc., New Brunswick, NJ, USA) and D06041501 rodent diet containing 10 kcal% fat (Research Diets, Inc.). The two groups were fed the high-fat diet for 12 weeks to establish the NAFLD model [42,43]. By using the same method, we first carried out pre-experiments. Considering the toxicology and the dose conversion relationship between human and mouse in previous reports [44,45], three concentration gradients (62.5 mg/kg, 250 mg/kg, and 1000 mg/kg) were investigated, and the dose of 250 mg/kg was proved to be effective and to have the least side effect. One of the high-fat groups was subjected to gavage of AEN (250 mg/kg·bw, dissolved in 0.5% CMC) for 4 weeks while maintaining the above diet. Two other groups of mice were treated with solvent 0.5% CMC. After euthanasia of the mice, blood and tissue samples were stored at −80 °C until further analysis.

### 2.4. Serum Biochemical Index Determination

The mouse blood was placed in a sterile EP (Eppendorf) tubes and stored at room temperature (25 °C) for 2 h. The serum was separated by centrifugation (3500 rpm, 10 min) and transferred to new sterile tubes. All of the serum samples were stored at −80 °C. Serum triglycerides (TG), total cholesterol (TC), low-density lipoprotein cholesterol (LDL-c), high-density lipoprotein cholesterol (HDL-c), and hemoglobin A1c (HbA1c) in glycosylated hemoglobin (GHb) were measured using an automatic biochemical analyzer (Mindrayn BS-480). At the same time, the liver function index was determined from measures, such as alanine aminotransferase (ALT), aspartate aminotransferase (AST), alkaline phosphatase (ALP), glutamyl transpeptidase (GGT), and cholinesterase (CHE). In addition, fasting blood glucose (FBG) and oral glucose tolerance (OGT) were measured using a blood glucose meter (Roche, USA).

### 2.5. Hematoxylin and Eosin (H&E) and Oil Red O Staining

Fresh liver tissue was collected and divided into three parts. The tissue was freshly fixed with a 4% paraformaldehyde solution. After fixing tissues, we used alcohol and xylene for gradient dehydration. After dehydration, the fixed tissues were treated with the paraffin-embedded method. The tissue section was stained with hematoxylin and eosin (H&E). For oil red O staining, fresh liver tissues were quickly frozen in liquid nitrogen. Part of the frozen liver specimens was embedded in optimum temperature cutting compound, cut into 6–8 μm sections, and stained with oil red O and hematoxylin and eosin. The tissue section was scanned using a digital slice scanner (Pannoramic MIDI II, 3DHISTECH Ltd., Budapest, Hungary) and analyzed by Image-Pro Plus software, and the area of hepatic steatosis was calculated using optical density values. The rest of the tissue was preserved at −80 °C.

### 2.6. Fatty Acid Analysis of Tissue Samples

The following samples were taken and freeze-dried in a vacuum freeze dryer (Labconco, Kansas City, MO, USA): 30 mg each of liver tissue, adipose tissue, and 25 μL of mouse blood and high-fat feed. Each was homogenized with 500 μL methanol before lipid extraction. Fatty acid methyl esters (FAME) were prepared by centrifuging at 4 °C at 1000 rpm for 5 min, then for 5 min with 2 mL 0.5 M sodium hydroxide methanol solution at 100 °C, after which 2 mL of 14% boron trifluoride methanol solution (w/w) was added and vortexed for 5 min [46,47]. FAME was extracted with hexane, and 1 μL of the solution was analyzed on a gas chromatograph (GC2010 plus, Shimadzu, Kyoto, Japan) fitted with a QP2010 ultra mass spectrometer (Shimadzu, Kyoto, Japan) using an Rtx-WAX column (30 m × 0.25 mm i.d. with 0.25 μm thickness, Restek Corporation, Bellefonte, PA, USA). Temperature programming for the gas chromatography was performed, as described previously [36]. The sample peak of FAME was identified by comparing the retention times obtained with pure FAME standards (Sigma, Shanghai, China).

### 2.7. Quantitative Real-Time Polymerase Chain Reaction (qRT-PCR) Analysis

Total RNA was isolated from the cells and liver tissue using TRIzol (Invitrogen, Carlsbad, CA, USA). LO2 cells were plated in 6-well plates and treated with AEN at different doses (25, 50, and 100 µg/mL). After 48 h, the culture medium was discarded, and the cells were washed and mixed with TRIzol for RNA extraction. RAW 264.7 cells were plated in 6-well plates and treated with AEN (100 µg/mL), FFA (oleic acid:palmitic acid = 2:1), and AEN + FFA. After 48 h, the culture medium was discarded, and the cells were washed and mixed with TRIzol. The mouse liver tissue was ground using a high-throughput tissue grinder (XinZhi, Zhejiang, China) before adding TRIzol. Complementary DNA (cDNA) was generated with 1 μg of total RNA using a RevertAid first-strand cDNA synthesis kit (Thermo Fisher, Waltham, MA, USA) following the manufacturer’s instructions. Qualitative analysis of the gene expression was performed using reverse-transcription PCR and agarose gel electrophoresis. A quantitative real-time polymerase chain reaction was also performed with the Bio-Rad CFX Connect Real-Time System (Bio-Rad, Hercules, CA, USA). The results were analyzed by qRT-PCR using the 2^−ΔΔCt^ method.

### 2.8. Cytokines Measurements

The fresh livers from mice were homogenized at a 1:10 (*m/v*) dilution in radioimmunoprecipitation assay (RIPA) lysis buffer (Beyotime, Nanjing, China). The levels of various cytokines that reflect the severity of inflammation, such as (TNF)-α, interleukin (IL)-1β, IL-6, and IL-10, in these homogenates were then determined using enzyme-linked immunosorbent assay (ELISA) kits (R&D, Minneapolis, MN, USA).

### 2.9. Western Blotting

The liver tissues were lysed with RIPA buffer containing protease inhibitors (Beyotime, Shanghai, China) and centrifuged at 4 °C for 15 min at 5000 g. Subsequently, the supernatants were collected. Equal amounts of protein, determined by a BCA protein assay (BCA protein assay kit, Beyotime, Shanghai), were separated using a 10% SDS-polyacrylamide gel. After SDS-PAGE, the proteins were transferred to a polyvinylidene fluoride membrane following the manufacturer’s instructions. The membrane was blocked with 5% (wt/vol) skim milk in Tris-buffered saline (TBS)/Tween 20 for 1 h at room temperature and incubated overnight at 4 °C with FASN antibody and β-actin (Cell Signaling Technology, MA, USA), SREBP-1c, and AMPK/p-AMPK (Abcam, MA, USA). This was followed by incubation with peroxidase-conjugated anti-rabbit IgG antibody (Sigma, St Louis, MO, USA) in a blocking solution according to the manufacturer’s instructions and then visualization with a chemiluminescence reagent (Amersham Bioscience, Piscataway, NJ, USA). Based on an analysis of the gray intensity, the semi-quantitative protein expression was analyzed using AlphaView software.

### 2.10. Chemical Composition Analysis of AEN Using GC-MS

The chemical composition of AEN was determined using a Hewlett-Packard 5890 II GC (Ramsey, MN, USA) equipped with an RP-5 MS capillary column (30 m × 0.20 mm, film thickness 0.22 μm) and an HP5972 mass-selective detector for the separation. Helium was the carrier gas at a flow rate of 1.5 mL min^−1^, and 1 μL of AEN was injected using a 1:10 split ratio. The column was initially programmed at 80 °C for 4 min and increased to 320 °C at a rate of 20 °C min^−1^, where it was held for 20 min. The electron energy and ion source temperature of the mass chromatography were 70 eV and 300 °C, respectively. The components were identified by comparing their GC retention indexes, using the NIST mass spectral search program (version 2.0, National Institute of Standards and Technology) and mass spectra with published data.

### 2.11. Statistical Analysis

All of the data were presented in the form of mean ± SD. The differences between variants were analyzed using Student’s *t*-test (Graph Pad Prism 6) for unpaired data. Then, the normality test (Shapiro–Wilk test) was used to decide if the sample fit the normal distribution by Tukey’s (SPSS 19.0). The data from the animal experiments were analyzed by one-way analysis of variance (ANOVA) by Tukey’s (SPSS 19.0). Values of *p* < 0.05 were considered statistically significant.

## 3. Results

### 3.1. Preliminary Component Identification

**Figure 1:**
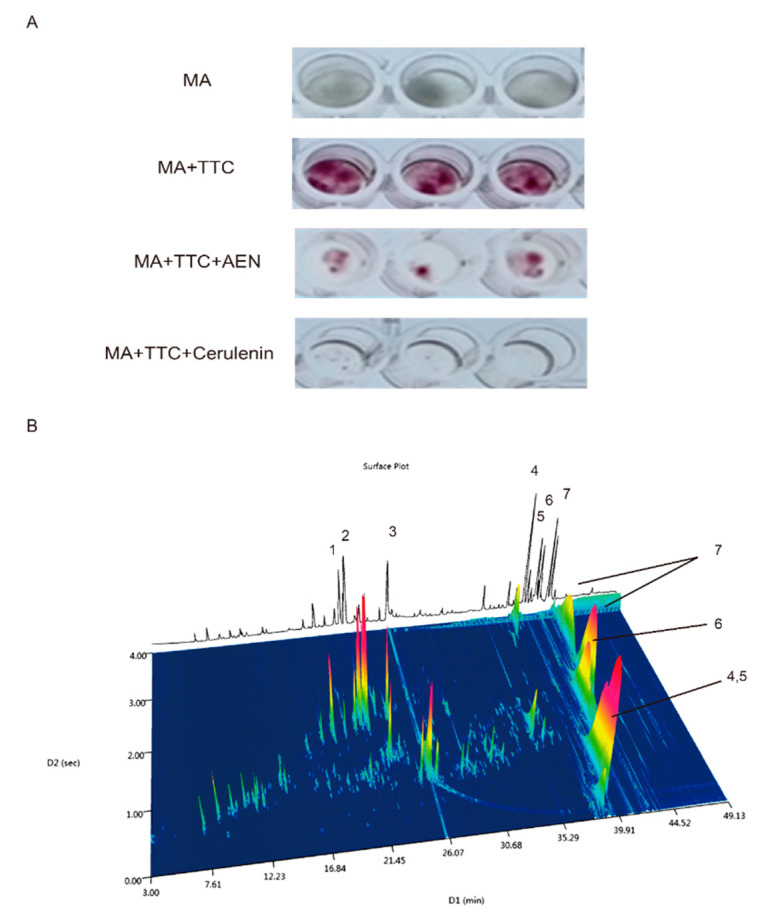

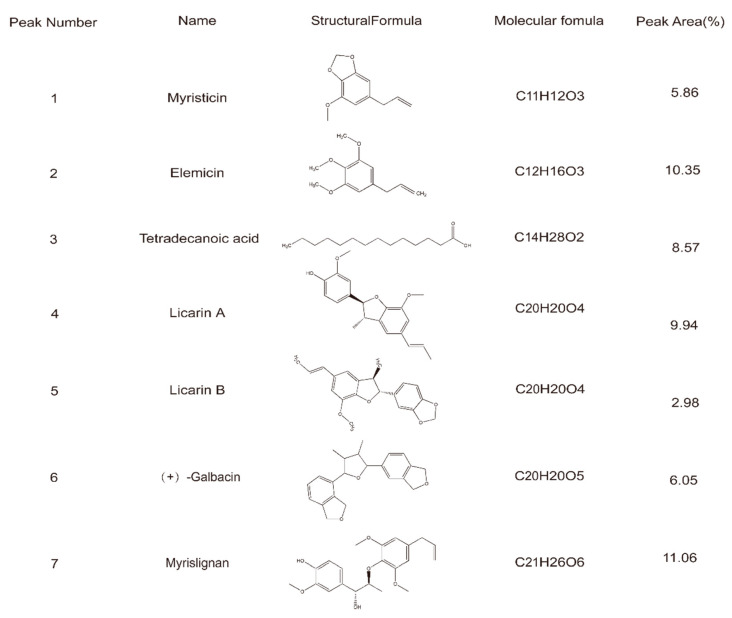
Screening and identification of alcohol extract of nutmeg (AEN). (**A**) Lipid synthesis inhibition screening using *Mortierella alpina* ATCC32222 (MA). There were four groups: the first three wells contained *M. alpina* (MA), the next three contained MA and TTC (2, 3, 5-triphenyltetrazolium chloride, 0.05%), the following three contained MA, TTC, and AEN (nutmeg extract), and the last three contained cerulenin (1 μM) as a positive control for lipogenesis inhibition. (**B**) Volatiles identified in AEN: comprehensive two-dimensional gas chromatogram of AEN by GC × GC-TOF-MS and general information: 1 myristicin, 2 elemicin, 3 tetradecanoic acid, 4 licarin A, 5 licarin B, 6 (+)-galbacin, 7 myrislignan.

In a previous study, we reported on the screening of consumable herbaceous plants for lipid synthesis inhibitors based on the degree of TTC (2, 3, 5-triphenyltetrazolium chloride) staining of *Mortierella alpina* as a marker for lipid content. Using the same approach in this study, we found that the alcohol extract of nutmeg could inhibit lipid production (Figure 1A) and further verified its ability to inhibit fatty acid synthesis in hepatocytes.

We then analyzed the chemical composition of AEN by GC × GC-TOF-MS. The results showed seven major compounds (relative content positive >2%; negative matching degree >800) in the nutmeg extract: myristicin (5.86%), elemicin (10.35%), tetradecanoic acid (8.57%), licarin A (9.94%), licarin B (2.98%), (+)-galbacin (6.05%), and myrislignan (11.6%) (Figure 1B).

### 3.2. AEN Inhibited Gene Expression for Fatty Acid Synthesis and Inflammatory In Vitro

**Figure 2:**
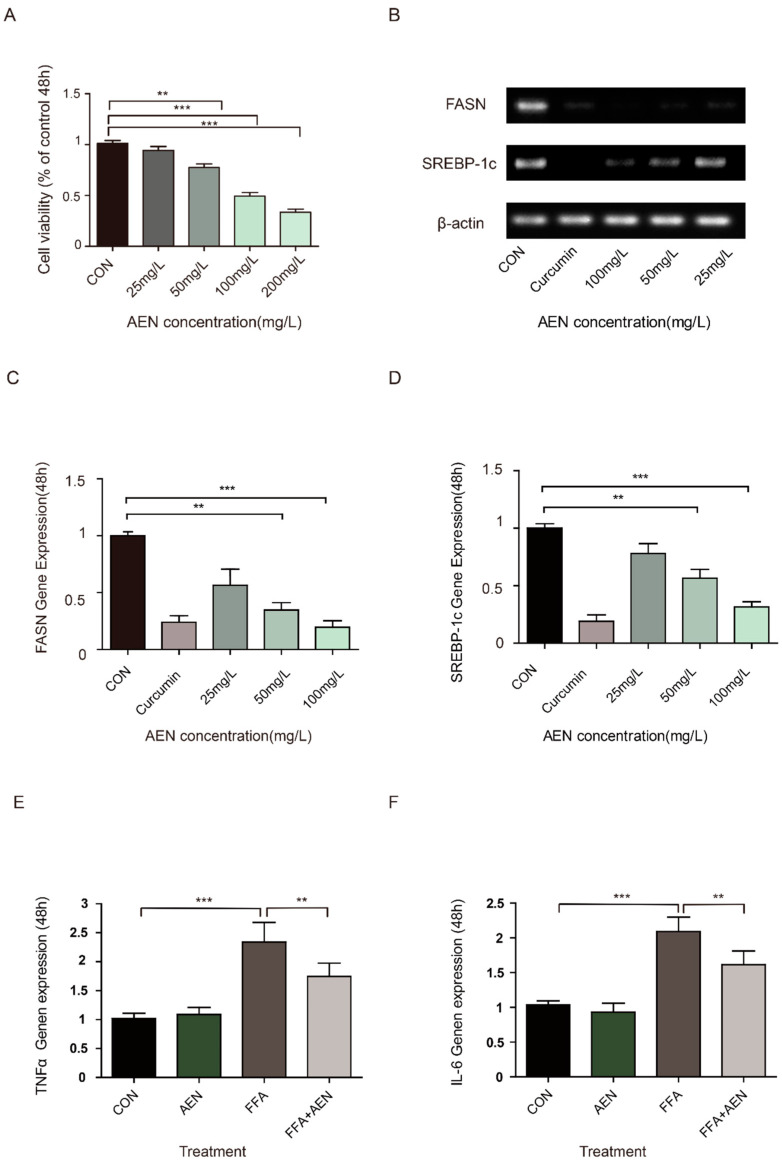
Alcohol extract of nutmeg (AEN) downregulated fatty acids and inflammation in cells. (**A**) Different concentrations of AEN (25 µg/mL, 50 µg/mL, 100 µg/mL, and 200 µg/mL) affected the cell viability of LO2 cells in a concentration-dependent manner. (**B**–**D**) The effects of different concentrations of AEN (25 µg/mL, 50 µg/mL, 100 µg/mL, and 200 µg/mL) on fatty acid synthase gene (FASN) and sterol regulatory element-binding protein-1c (SREBP-1c) gene expression after 48 h treatment in LO2 cells. (**E**,**F**) 100 µg/mL of AEN, free fatty acid (FFA) (oleic acid:palmitic acid = 2:1), and AEN + FFA on tumor necrosis factor α(TNF-α) and interleukin-6 (IL-6) gene expression after 48 h treatment in RAW264.7 cells. The mRNA expression was determined by RT-qPCR (*n* = 3). The gene expression with untreated cells was set to 1. Data are shown as mean ± SD. (**A**) The absorbance and proliferation of cells at 570 nm were measured by a microplate reader after 48 h treatment. The absorbance of untreated cells was set to 1. Data are shown as mean ± SD of the six replicate wells representative of three independent experiments. ** *p* < 0.01, *** *p* < 0.001.

We used an MTT assay on LO2 cells to determine the optimum concentration and time for effective AEN treatment. After 48 h of AEN treatment, the survival rate of LO2 was about half that in the control group at a concentration of 100 µg/mL AEN (Figure 2A). To further demonstrate that AEN can inhibit lipid synthesis, we analyzed the expression of the FASN gene and SREBP-1c in LO2 cells. AEN treatment significantly decreased the gene expression of FASN and SREBP-1c in LO2 cells in a dose-dependent manner (Figure 2B–D). In particular, the inhibitory effect of AEN at a concentration of 100 µg/mL was the most significant, which was similar to the IC50 value obtained from the MTT assay. After 48 h treatment (Figure 2E,F), the mRNA expression of cytokines in RAW264.7 cells treated with AEN (100 µg/mL), FFA (oleic acid:palmitic acid = 2:1), and AEN + FFA was analyzed. Compared with the control group, TNF-α and IL-6 were both evidently increased in the FFA group, and there was no significant change in the AEN group. Although the upward trend of inflammation was mitigated in group AEN + FFA, there were still significant differences compared with the control group.

### 3.3. AEN Treatment Reduced Obesity and Blood Glucose in Mice Induced by a High-Fat Diet

**Figure 3:**
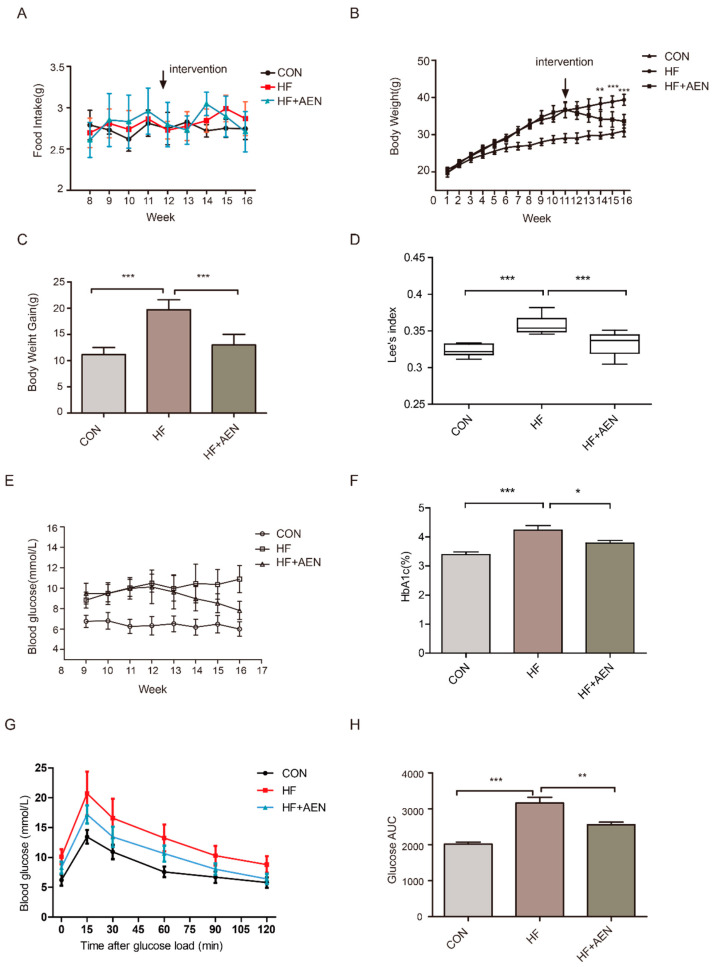
AEN alleviated obesity and blood glucose levels in high-fat diet-induced nonalcoholic fatty liver disease (NAFLD) mice (C57/bl6). (**A**) Changes in average daily food intake of each mouse in the three groups: HF (high fat diet), HF + AEN, and conventionally raised CON (normal control) mice from the 8th to the 16th week. (**B**) Mouse body weight growth in the three groups (HF, HF + AEN, and CON) for 16 weeks (*n* = 10). Data are shown as mean ± SD. (** *p* < 0.01, *** *p* < 0.001). (**B**) AEN intervention began in the 12th week. (**C**) Bodyweight gain of CON, HF, and HF + AEN groups. For each group, *n* = 10. Data are shown as mean ± SD. (*** *p* < 0.001). (**D**) The difference in Lee’s index among the three groups (CON, HF, and HF + AEN). Lee’s index: 1000 * body weight (g) ^ (1/3)/body length (body length is defined as the distance from nose to anus). (***p < 0.001). (**E**) The variation in fasting blood glucose levels in CON, HF, and HF+AEN fed mice from the 9th to the 16th week. (**F**) Changes in glycosylated hemoglobin (HbA1c) in the mice over 16 weeks. For each group, *n* = 10. Data are shown as mean ± SD (* *p* < 0.05, *** *p* < 0.001). (**G**) The oral glucose tolerance test (OGTT) showed fasting blood glucose for each group of mice and the changes after oral glucose at different time points. For each group, *n* = 10. Data are shown as mean ± SD. (**H**) The area under the blood glucose concentration-time curve (AUC) according to the OGTT curve. (** *p* < 0.01, *** *p* < 0.001).

There was no significant difference in food intake between the two experimental groups (HF (high fat diet) and HF + AEN) (Figure 3A). In terms of weight, from the 15th week of the experiment (after 3 weeks of oral supplementation with AEN), the bodyweight of the mice in the HF + AEN group changed significantly compared with that of the HF group (Figure 3B,C). Lee’s index (Lee’s index = body weight (g) ^ (1/3) × 1000/body length (cm)) was applied to further measure the degree of obesity of the mice. Compared with the HF group, there was a significant decrease in the AEN treatment group (Figure 3D). The fasting blood glucose of the mice was measured once a week from the 9th week (Figure 3E). The average blood glucose level of the high-fat diet (HFD) mice was higher (Glu ≥ 10 mmol/L) than that of the control group (Glu < 7 mmol/L). After 4 weeks of treatment with AEN, the fasting blood glucose level of the AEN group was significantly downregulated to below 8 mmol/L. Blood glycated hemoglobin in the treatment group showed some improvement in the 16th week after 4 weeks of AEN treatment (Figure 3F). According to the OGT test at the 15th week, glucose tolerance was damaged in the HF group (early symptoms of diabetes mellitus), whereas there was significantly increased glucose tolerance in the AEN treatment group (Figure 3G). We corroborated this result by calculating the area under the blood glucose concentration-time curve (AUC) (Figure 3H). These data suggested that oral supplementation with AEN could reduce the body weight and blood glucose levels of obese mice and improve obesity-related symptoms.

### 3.4. AEN Improved Hepatocyte Steatosis and Liver Function in Mice

**Figure 4:**
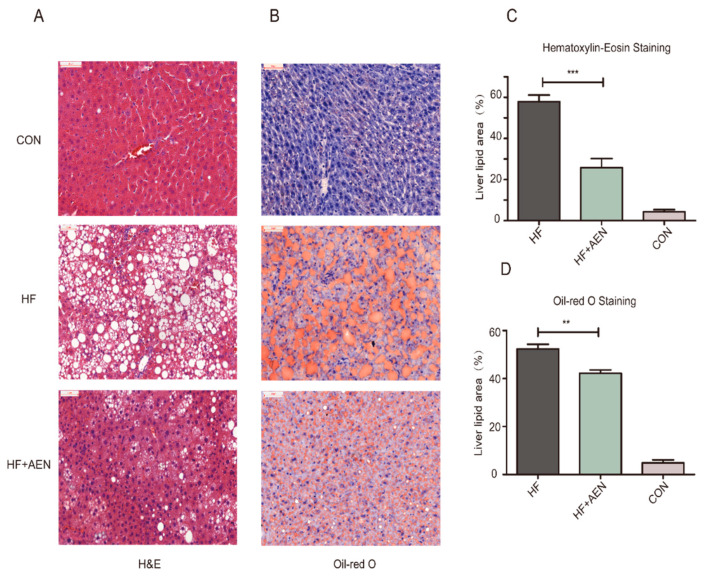

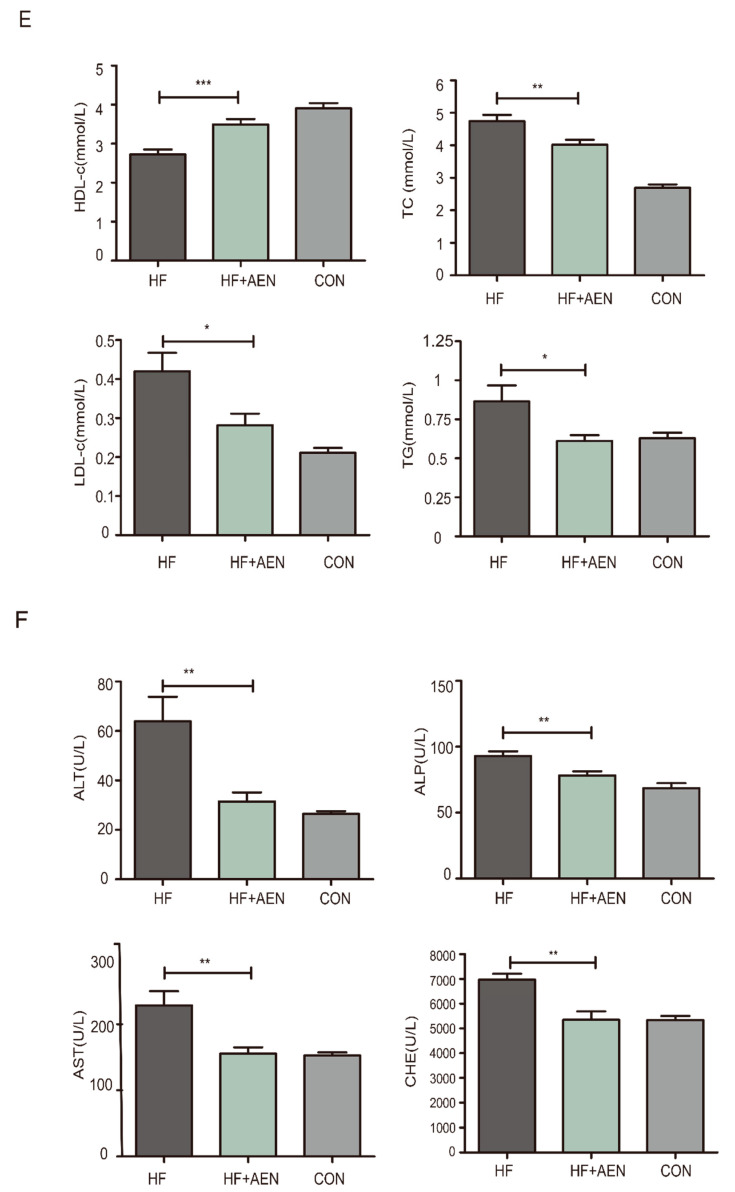
AEN alleviated hepatocyte steatosis, blood lipids, and liver function in mice. (**A**) Hematoxylin and eosin staining of liver sections. (**B**) Oil red O and hematoxylin staining of liver sections. (**C**,**D**) The liver lipid area of H&E, oil red O, and hematoxylin-stained sections of HF, HF + AEN, and CON groups was measured and calculated by Image-Pro Plus software (*n* = 5 per group). Data are shown as mean ± SD. (**p < 0.01, *** *p* < 0.001). (**E**) Effect of AEN on four types of blood lipids in the high-fat diet (HFD)-induced NAFLD mice: HDL-c (high-density lipoprotein), LDL-c (low-density lipoprotein), TC (total cholesterol), and TG (triglycerides) were measured in mouse serum samples. (**F**) Effect of AEN on four items of the liver function index in HFD-induced NAFLD mice: ALT (alanine aminotransferase), AST (aspartate aminotransferase), ALP (alkaline phosphatase), and CHE (cholinesterase). Data are shown as mean ± SD (*n* = 10 per group, * *p* < 0.05, ** *p* < 0.01, *** *p* < 0.001).

A high-fat diet can not only cause obesity but also induce fatty liver formation. As the liver is important for lipid metabolism, the accumulation of excessive lipids in the liver can accelerate the progression of NAFLD. We studied and analyzed the possibility that AEN could reduce the development of NAFLD and relieve its symptoms. At the end of the 16th week, the mice were euthanized, and the NAFLD-related physical indexes, liver function, glucose, and lipid metabolism were analyzed. The mouse liver sections were used to analyze liver lipid accumulation and the degree of hepatocyte steatosis (Figure 4). Compared with the serious hepatocellular fatty degeneration in the HF group, diffused hepatocytes steatosis with lipid vacuolation, inflammation, and ballooning degeneration (marked with arrows in the figure) were ameliorated in the AEN treatment group (HF + AEN), as shown by H&E staining (Figure 4A,B). While fat droplets accounted for more than 50% of the liver area in the HF group (a typical NAFLD stage), they accounted for less than 10% in the normal CON group. The liver fat droplet area of the AEN treatment group had been reduced to varying degrees with both staining methods, demonstrating that AEN could effectively reduce hepatic steatosis (Figure 4C,D). The analysis of serum biochemical indexes and liver function indexes showed that AEN could effectively increase high-density lipoprotein (HDL-c), decrease total cholesterol (TC) levels, and downregulate triglyceride (TG) and low-density lipoprotein (LDL-c) levels to an extent (Figure 4E). There was a significant decrease in the liver function indexes closely related to hepatic adipose infiltration and hepatitis: ALT (alanine aminotransferase), AST (aspartate aminotransferase), ALP (alkaline phosphatase), and CHE (cholinesterase) (Figure 4F). These results suggested that AEN reduced hepatocyte steatosis and its associated liver function indexes, such as ALT and AST.

### 3.5. AEN Regulated Fatty Acid Metabolism and Inflammatory Reaction in Mice

**Figure 5:**
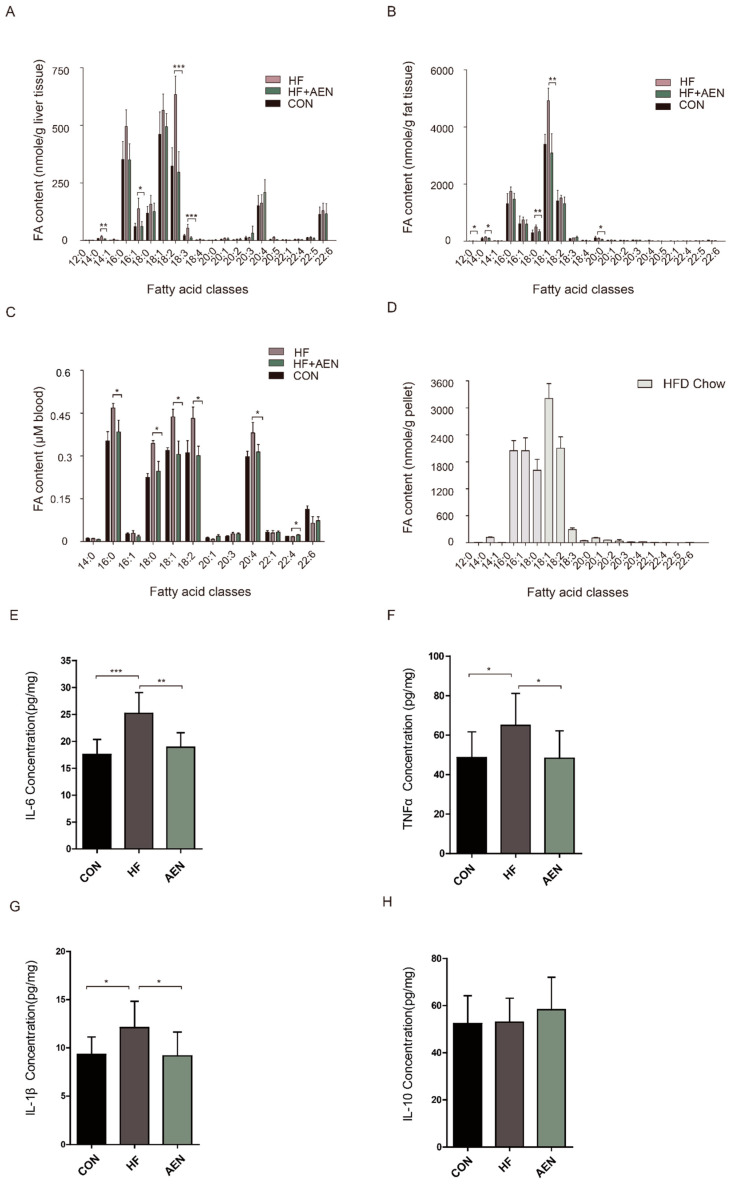
AEN regulated fatty acid species and contents and liver inflammation in NAFLD mice. FFA species and contents were quantified in (**A**) mouse liver, (**B**) white fat, and (**C**) blood samples of HF, HF + AEN, and conventionally raised (CON) mice. The FFAs were quantified in the HF pellets (**D**). AEN regulated cytokines TNF-α (**E**), IL-6 (**F**), IL-1β (**G**), and IL-10 (**H**) in mice liver. Data shown are averages of *n* = 8 mice. Error bars represent 1 SD. * *p* < 0.05, ** *p* < 0.01, *** *p* < 0.001.

AEN not only inhibited lipid synthesis in cells in vitro but also slowed down the liver lipid accumulation in mice in vivo. The analysis of free fatty acid (FFA) species confirmed a significant increase in both saturated and unsaturated fatty acids in the liver, epididymal fat, and blood of the HF mice compared with the control mice. AEN treatment reduced both saturated and unsaturated fatty acids to varying degrees (Figure 5). In the liver, saturated fatty acids, such as myristic acid, and unsaturated fatty acids, such as palmitoleic, oleic, and α-linolenic acid, decreased significantly in the AEN group. Hexadecanoic acid and oleic acid had downward trends, but it was not significant. The growth of arachidonic acid was not obvious (Figure 5A). Compared with the HF group, oleic acid and most saturated fatty acids (except for palmitic acid) in epididymal fat were decreased in HF + AEN group (Figure 5B). In blood, palmitic acid and the C18 (18:0–18:2) fatty acid family decreased, whereas individual polyunsaturated fatty acids (PUFAs), such as docosatetraenoic acid, increased (Figure 5C). The FFAs quantitatively dominant in the diet were palmitic, palmitoleic, stearic, oleic, and linoleic acids (Figure 5D). With the exception of arachidonic (ARA) and docosahexaenoic (DHA) acid in the liver and blood, these were also the five most abundant FFAs in all three of the tissues examined. Inflammatory response and accumulation of fatty acids were closely related. Thus, we measured the inflammatory cytokines in the liver (Figure 5E–H). Compared with the control group, pro-inflammatory factors, such as TNF-α, IL-6, and IL-1β, were significantly downregulated (Figure 5E–G). However, there was no significant change in IL-10, an inflammation and immunosuppressive factor (Figure 5H).

**Figure 6:**
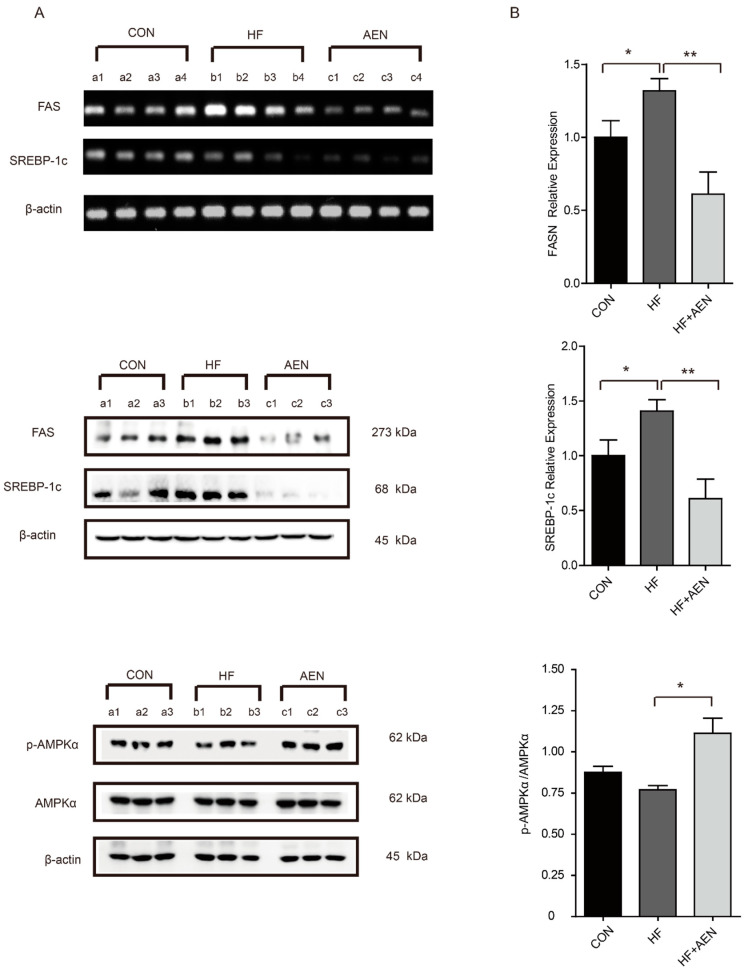
AEN could regulate the AMP-activated protein kinase (AMPK)-mediated SREBP signaling pathway in fatty liver in mice induced by a high-fat diet. (**A**) Agarose gel electrophoresis of FASN and SREBP-1c mRNA expression in mouse liver, with β-actin as housekeeping gene (*n* = 4). Western blot analysis of FASN, SREBP-1c, AMPKα, and p-AMPKα protein expression in mouse liver, with β-actin as a housekeeping protein (*n* = 3). (**B**) Band analysis of the relative expression in the mouse liver of FASN, SREbBP-1c, and p-AMPKα/AMPKα proteins normalized to β-actin (*n* = 3). The protein expression of the CON group was set to 1. Data are shown as mean ± SD. Different letters show significant differences (* *p* < 0.05, ** *p* < 0.01).

We further studied the potential mechanisms for the decrease in fatty acids in these tissues (Figure 6). AEN treatment decreased the mRNA expression of FASN and SREBP-1c in the NAFLD mouse liver. We verified that this change also occurred at the protein level in mouse fatty liver. The expression and phosphorylation of AMPKα as the upstream regulators of SREBP-1c were found to be related to the regulation of AEN. In other words, AEN further inhibited the expression of downstream FASN and SREBP-1c by activating the phosphorylation of AMPKα (Figure 6A). The relative expression of FASN, SREBP-1c, and p-AMPKα/AMPKα proteins was calculated by the gray values or optical density analysis (Figure 6B). These results suggested that the regulation of fatty acid synthesis by AEN was realized by regulating targets related to lipid metabolism.

## 4. Discussion

The potential sources of fatty liver include dietary fatty acids from chylomicrons and lipids synthesized in de-novo lipogenesis (DNL) or from plasma free fatty acids [48]. Thus, fatty acid uptake and fatty acid synthesis disorders may affect lipid metabolism in the liver [49]. Accompanied by excess fatty acid intake, a high-fat diet can lead to obesity, NAFLD, and other metabolic syndromes [50,51]. In this study, by using the NAFLD model induced with a high-fat diet, we observed the increase of body weight and levels of harmful blood fats. Moreover, the data for the pathological section showed diffused hepatocyte steatosis with lipid vacuolation, inflammation, and ballooning degeneration in the NAFLD mice. A significant increase in pro-inflammatory cytokines (TNF-α, IL-6, and IL-1β) in the high-fat diet-induced mice also proved data of the pathological section above. Fatty acids can be seen to play a key role in the formation of NAFLD: On the one hand, dietary fats are fully hydrolyzed, releasing FFAs, approximately 20% of which are delivered to the liver [52]; on the other hand, the accumulation of free fatty acids in hepatocytes can produce lipotoxicity, resulting in inflammatory reactions and hepatocyte injury [53,54]. To achieve a balance between liver and blood circulation, the liver absorbs more FFAs into the hepatocytes and adds a burden to liver functioning [55]. AEN effectively downregulated the FFAs in the liver, fat, and blood of the NAFLD mice. In the serum biochemical index, with the improvement of hyperlipidemia, the liver function recovered.

We proved that AEN downregulated FFAs-induced pro-inflammatory cytokines in vitro, and this inhibition was associated with fatty acid regulation. Our data also indicated that AEN effectively regulated the expression of related factors (FASN and SREBP-1c) in the fatty acid metabolic signaling pathway, both in vivo and in vitro. From this, we inferred that AEN might be the key to lipid regulation and inhibition of inflammatory response. Increased de novo synthesis can lead to further fatty acid accumulation in hepatocytes. The process of liver synthesis of endogenous FAs includes the de novo synthesis of FFAs through a complex cytosolic polymerization in which glucose is converted to acetyl-CoA by glycolysis and the oxidation of pyruvate [56,57]. In the process, FASN and SREBP-1c make prominent contributions. FASN catalyzes the formation of palmitic acid from malonyl-CoA and acetyl-CoA in hepatocytes [58]. Previous studies in our laboratory have shown that the inhibition of FASN can alleviate obesity in mice [24]. This was similar to the effect of AEN in reducing body weight and fat accumulation in NAFLD mice. A previous study has proved that fatty acid synthesis by FASN is tightly regulated by SREBP-1c and that its expression is also regulated negatively by AMP-activated protein kinase (AMPK) and positively by insulin [59]. In this study, AEN activated AMPKα phosphorylation and downregulated the expression of downstream SREBP-1c and FASN proteins and genes. AEN not only inhibits fatty acid synthesis but also regulates fatty acid composition. AEN can reduce the proportion of fatty acids, especially saturated fatty acids, in liver, fat, and blood. Saturated fatty acids (C16:0, C18:0) can bring liver injury with hepatic steatosis and lipid-induced apoptosis in hepatocyte [60,61]. In addition, AEN decreased blood glucose levels and improved glucose tolerance in the mice. We assumed that elevated blood glucose in mice led to an insulin response, which activated the expression of SREBP-1c and FASN, and that AEN reversed this trend. However, we need to further prove the cause of high blood sugar and its relationship with fatty acid regulation. 

The components in *Myristica fragrans Houtt.* also have some other reported functions. It has been reported that nutmeg is potentially valuable as an edible plant for its role in lipid metabolism [36,37]. By improving lipid metabolism, excessive fatty acid intake and storage imbalance are adjusted. There is a kind of natural dietary fatty acids named tetradecanoic acid (myristic acid) in AEN. Dietary myristic acid increases the HDL-cholesterol concentration and shows a protective effect on atherosclerosis [62]. It is reported that the oral administration of myristic acid improves hyperglycemia by improving glucose tolerance and reducing body weight [58]. Myristicin belongs to the methylenedioxyphenyl or allyl-benzene family of compounds, which are found widely in parsley, carrots, black pepper, and nutmeg [27]. Morita et al. found that myristicin protected rats against liver damage caused by lipopolysaccharides and d-galactosamine [63], but its effect on liver lipid metabolism needs to be further verified. Shyni et al. determined that licarin B improved insulin sensitivity via PPARγ and activation of glucose transporter 4(GLUT4) in the IRS-1/PI3K/AKT (insulin receptor substrate-1/phosphoinositide 3-kinase/protein kinase B) pathway in 3T3-L1 adipocytes [64]. Elemicin is an important alkaloid, widely used as a spice and raw material, and can be used in the synthesis of mescaline, confectionery, condiments, non-alcoholic beverages, etc. [65]. Elemicin has the potential of inhibiting pro-inflammatory cytokines, which are considered to be an effective therapeutic approach for the treatment of epileptic disorders [66]. Myrislignan, one of the lignans, has the potential to alleviate TAA-induced liver injury through the modulation of PPAR-α [37]. This indicates a potential candidate for the study of myrislignan on liver lipid metabolism in mice. In the lignans family, the function of licarin A and (+)-galbacin has been less reported. These will need to be further gone into. Furthermore, dietary myristic acid is also reported to be able to increase the tissue content of C20:5 n-3 (eicosapentaenoic acids, EPA) and C20:3 n-6 (polyunsaturated fatty acids, PUFA), which are essential fatty acids for human health [67].

## 5. Conclusions

Our experimental results showed that AEN could effectively alleviate high-fat diet-induced obesity and NAFLD in mice. It could also significantly reduce the accompanying symptoms of obesity and hyperglycemia. Liver function and blood lipid levels were simultaneously improved. AEN downregulated pro-inflammatory level in the liver of mice. Moreover, AEN could inhibit the expression of FASN and SREBP-1c through the activation/phosphorylation of AMPKα in hepatocytes, both in vivo and in vitro. Our results strongly suggested the need to further explore the mechanism by which AEN alleviates NAFLD and the effective functional components.
